# Light-emitting self-assembled metallacages

**DOI:** 10.1093/nsr/nwab045

**Published:** 2021-03-17

**Authors:** Jun Zhao, Zhixuan Zhou, Guangfeng Li, Peter J Stang, Xuzhou Yan

**Affiliations:** School of Chemistry and Chemical Engineering, Frontiers Science Center for Transformative Molecules, Shanghai Jiao Tong University, Shanghai 200240, China; Department of Chemistry, University of Utah, Salt Lake City, UT 84112, USA; School of Chemistry and Chemical Engineering, Frontiers Science Center for Transformative Molecules, Shanghai Jiao Tong University, Shanghai 200240, China; Department of Chemistry, University of Utah, Salt Lake City, UT 84112, USA; School of Chemistry and Chemical Engineering, Frontiers Science Center for Transformative Molecules, Shanghai Jiao Tong University, Shanghai 200240, China

**Keywords:** supramolecular coordination complexes, coordination-driven self-assembly, aggregation-induced emission, metallacages, organometallic materials

## Abstract

Coordination-driven self-assembly of metallacages has garnered significant interest because of their 3D layout and cavity-cored nature. The well-defined, highly tunable metallacage structures render them particularly attractive for investigating the properties of luminophores, as well as for inducing novel photophysical characters that enable widespread applications. In this review, we summarize the recent advances in synthetic methodologies for light-emitting metallacages, and highlight some representative applications of these metallacages. In particular, we focus on the favorable photophysical properties—including high luminescence efficiency in various physical states, good modularity in photophysical properties and stimulus responsiveness—that have resulted from incorporating ligands displaying aggregation-induced emission (AIE) into metallacages. These features show that the synergy between carrying out coordination-driven self-assembly and using luminophores with novel photophysical characteristics like AIE could stimulate the development of supramolecular luminophores for applications in fields as diverse as sensing, biomedicine and catalysis.

## Teaser

Incorporation of luminophores with novel photophysical characteristics, such as aggregation-induced emission, to supramolecular coordination cages represents a versatile strategy for developing supramolecular luminophores with emerging functionalities and diverse applications.

## INTRODUCTION

Molecular self-assembly is a ubiquitous process in nature and involves molecules with predefined recognition elements spontaneously forming well-defined ensembles through noncovalent interactions such as hydrogen bonds, van der Waals forces, hydrophilic and hydrophobic interactions, and coordination interactions [1,2]. Inspired by the elegant self-assembly processes found in nature, many efforts have been devoted to designing and synthesizing novel supramolecular ensembles for practical applications. Coordination-driven self-assembly, which relies on the spontaneous formation of coordination bonds between Lewis-acidic ‘acceptor’ and Lewis-basic ‘donor’ molecular building blocks, has evolved into a powerful methodology for carrying out rational, predesigned self-assemblies of discrete supramolecular coordination complexes (SCCs) [3–5]. Functional SCCs can be readily prepared by introducing functional groups through pre- or post-self-assembly modifications [6–8]. Out of the plethora of reported SCCs, metallacages have shown particularly significant potential for various applications because of their favorable inherent properties. First, the modular nature of coordination self-assembly enables the precise spatial control over multiple and hetero-functional groups within the 3D supramolecular architecture, promoting new properties as a result of the synergy between the functional groups. Second, the presence of cavities in metallacages facilitates host–guest chemistry and has inspired the development of separation, sensing, delivery and catalysis applications of metallacages [9,10].

The development of luminophores with favorable photophysical properties is the basis for their applications in the areas of chemical probing [11], biological sensing and imaging [12], optoelectronic materials [13] and stimuli-responsive materials [14]. Studies on light-emitting SCCs stem from the use of rigid organic molecules as the building blocks for coordination-driven self-assembly. Many of these molecules have large conjugated systems and are inherently photophysically active, thus endowing the resulting SCCs with light-emitting properties. After these properties were first recognized, experimental and theoretical investigations that elucidated the photophysics of these SCCs were then conducted [15,16]. These results have provided the basis for preparing novel light-emitting metallacages that utilize their unique, tunable 3D structures. The stoichiometries, spatial positions and orientations of luminophores can be precisely modified and controlled when they are incorporated as components of self-assembled metallacages. Such modularity is highly challenging to realize using conventional covalent synthesis, but is nevertheless a goal worth striving for as it would promote the formation of interactions between the various components of the metallacages that may lead to the emergence of features and consequent applications that are not obtainable for an unincorporated luminophore. In addition, the almost limitless structural versatility of metallacages provides modularity over the photophysical profiles of the incorporated luminophore. These benefits are exemplified by studies on metallacages comprising luminophores with aggregation-induced emission (AIE) character.

AIE is a photophysical phenomenon displayed by some luminophores containing freely rotating groups. These luminophores, often referred to as AIEgens, are weakly emissive or nonemissive in dilute solutions but become highly emissive upon aggregating. Discovered by Tang and colleagues in 2001 [17], this phenomenon is in sharp contrast to aggregation-caused quenching (ACQ), a photophysical phenomenon widely observed for conventional luminophores and in which luminescence efficiency significantly decreases in the aggregated state owing to excited-state interactions. Mechanistic studies on luminophores displaying AIE revealed that their low luminescence efficiency in dilute solutions could be attributed to the intramolecular motions of the freely rotating groups that nonradiatively dissipate the excitation energy. This decay pathway is inhibited in the aggregated state, and thus a higher luminescence efficiency is observed for the AIEgens [18]. Manipulation of the intramolecular motions of the rotor units in AIEgens can be achieved both physically (by adjusting viscosity, temperature or pressure) and chemically (by forming covalent or noncovalent bonds). In 2015, Huang, Stang and coworkers observed that incorporating AIEgens into self-assembled metallacages resulted in high luminescence efficiency levels in both dilute solutions and aggregated states, thereby bridging the gap between the distinct AIE and ACQ phenomena [19]. This uncommon photophysical phenomenon, in combination with the unique tunable structures of metallacages, has inspired further studies to be carried out on self-assembled AIE-active metallacages. They have emerged as new platforms for fabricating light-emitting materials for applications in molecular probing and sensing, energy conversion, bioimaging and fabricating theranostic agents [20–27].

In this review, we summarize the current developments of light-emitting metallacages. The strategies used to prepare such metallacages are discussed, as well as their properties and consequent applications, which are enabled by the formation of metallacages through metal–ligand coordination interactions. Subsequently, AIE-active metallacages are highlighted. We expect that the illustrations provided here of their designs and applications should create more interest in this field, stimulate additional ideas from researchers and further advance the development of light-emitting metallacages by the synergistic combination with AIE and other photophysical phenomena.

## NON-AIE-ACTIVE LIGHT-EMITTING METALLACAGES

Studies on light-emitting SCCs originated from the use of rigid organic molecules as the building blocks for coordination-driven self-assembly. Many of these molecules include large conjugated systems and are inherently photophysically active, thus endowing the resulting SCCs with light-emitting properties. These early results motivated experimental and theoretical investigators to elucidate the photophysics of these SCCs, and provide the basis for developing light-emitting metallacages that display emerging photophysical properties. To date, researchers have employed luminophores as donor [28] or acceptor building blocks [29], or encapsulated guest molecules [30,31] inside the cavity of the metallacages (Table 1). The formation of a 3D cage structure brings various molecular building blocks into proximity and thus facilitates their interactions. This feature can be exploited for energy transfer schemes, which are beneficial for sensing, delivery and catalytic applications. However, it may also introduce low-lying dark states that decrease the luminescence efficiency and decrease the lifetime of the excited state. As a result, careful selection and arrangement of the molecular building blocks for self-assembly reactions are required to obtain metallacages with desired properties.

**Table 1.** Constituent building blocks and cartoon representations of non-AIE-active light-emitting metallacages.

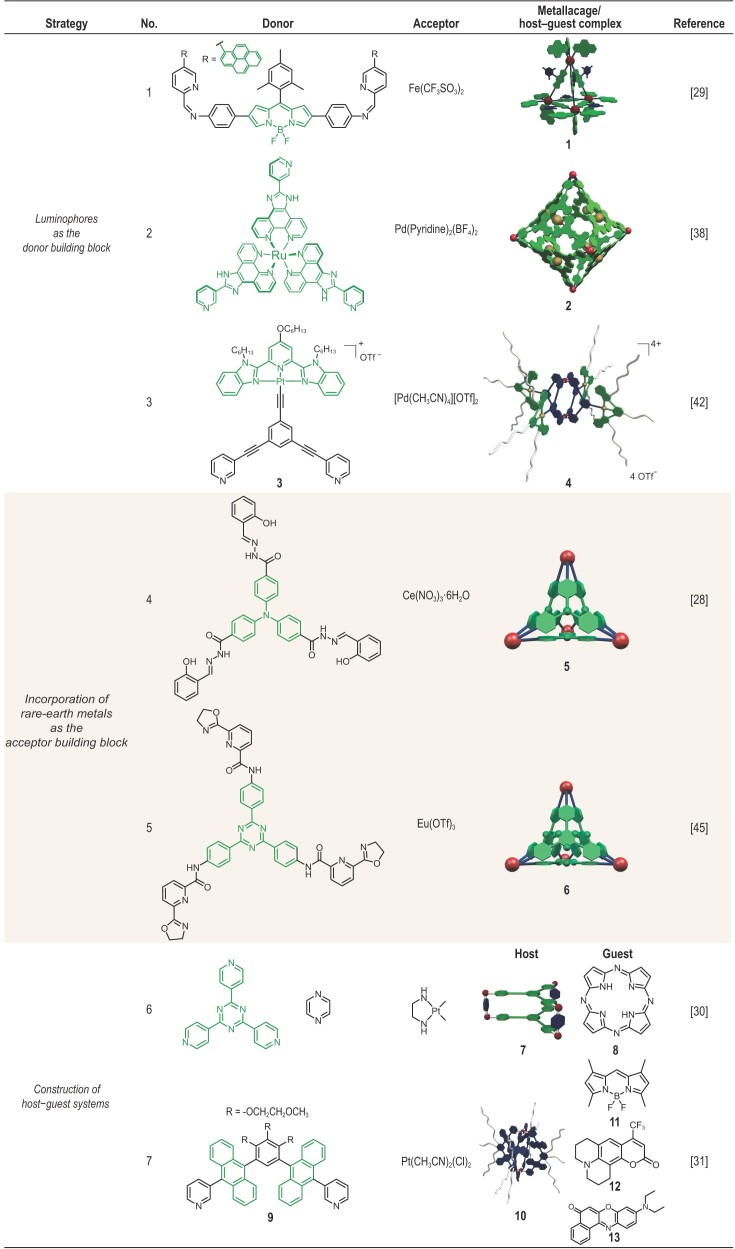

### Luminophores as the donor building blocks

As a consequence of their straightforward nature and well-established photophysical properties,

well-known luminophores are strongly considered for synthesizing light-emitting metallacages [32–34]. They are often modified with electron-rich recognition motifs and directly used as structural components for metallacages. Nitschke and coworkers selected and decorated widely used fluorescent dyes such as BODIPY and pyrene with diamine and aldehyde motifs to afford metallacage building blocks [29]. Combining them was found to form metallacage **1** via the simultaneous formation of both dynamic covalent and metal-coordination bonds with Fe(II). Metallacage **1** exhibited fluorescence originating from BODIPY with a quantum yield (Φ_F_) value of 1.5% in acetonitrile. Adding acetate to the solution of **1** was found to lead to a 28-fold enhancement in fluorescence, which was probably due to the binding of the acetate anion inside the cavity of the cage via electrostatic and anion–π interactions. Meanwhile, the presence of anion–π interactions could have perturbed the π orbitals of the ligand, which would explain the bathochromic shift of the absorption peak of **1**. Based on this guest responsiveness, Nitschke and coworkers further developed a white-light-emitting system by introducing perylene into the cavity of **1**, which was shown to emit white light with a Φ_F_ value of 11% and CIE coordinates of (0.30, 0.36).

In addition to organic luminophores, organometallic complexes have also been exploited as donor building blocks for self-assembling metallacages [35–37]. These complexes offered sophisticated structures and properties not displayed by pure organic ligands. Su and coworkers described a self-assembled Ru–Pd heterometallic coordination cage **2**, wherein the Ru-containing donor was shown to endow the metallacage with light-emitting character [38]. Metallacage **2** was found to exhibit an emission band peak at 610 nm, corresponding to the Ru-centered triplet metal-to-ligand charge transfer (MLCT) state. The intramolecular transfer of charge from Ru coordination complexes to Pd(pyridine)_4_ moieties enables metallacage **2** to be a light-harvesting system, endowing **2** with catalytic properties for photochemical hydrogen production.

Luminophores can also be introduced as pendant functional groups attached to the rigid structural components via covalent bonds [33,39–41]. Recently, Yang and coworkers prepared, via coordination-driven self-assembly, a new alkynylplatinum(II) bzimpy (**3**) containing metallacage **4**. This metallacage emitted light of different colors, from pale yellow to orange-red, when placed in mixtures of water/dimethylformamide (DMF) with different ratios [42]. Further investigation revealed this solvent-induced emission switch to be accompanied by an enhancement of the intensity of the emitted light as a result of changes in intermolecular Pt···Pt and π–π interactions.

### Incorporation of rare-earth metals as the acceptor building blocks

Electron-deficient metal ions or metal complexes are necessary structural elements for coordination-driven self-assembly. In many cases, they induce the formation of a significant number of low-lying dark states, such as those resulting from ligand-to-metal charge transfer and that perturb the photophysical properties of the luminophores within the metallacages. In contrast, lanthanides display inherent light-emitting properties such as specific emission wavelengths, large Stokes shifts due to their peripheral 4f electrons [43]. As a result, lanthanides are promising candidates for use in preparing light-emitting metallacages. A series of fluorescent molecular tetrahedrons were synthesized wherein the ligands with large absorption coefficients could act as efficient sensitizers for lanthanide ions via the ‘antenna effect’ [44]. In 2011, Duan, He and coworkers developed a neutral molecular tetrahedron **5**, composed of Ce^4+^ and triphenylamine ligands, and observed an emission band for **5** at a wavelength of 470 nm with a Φ_F_ value of 1% when excited at 350 nm in DMF [28]. Additionally, the well-defined cavity of **5** was shown to allow the encapsulation of 2-phenyl-4, 4, 5, 5-tetramethylimidazolineyloxyl-3- oxide (PTIO), a spin-labeling probe specific for NO only in living cells. The fluorescence of **5** could be quenched by PTIO but ‘turned on’ in the presence of NO. Therefore, this work presents an example of the development of light-emitting metallacages and exploring their potentials as bioimaging agents.

To prepare metallacages that display long-wavelength absorption and emission, which are favorable for biomedical applications, Sun, Han and coworkers developed a Eu^3+^ tetrahedral cage **6**, whose fluorescence could be induced by visible light through intraligand charge-transfer sensitization (ILCT) [45]. The electron-deficient triazine unit within the ligand leads to high photosensitization efficiency, thus enabling the photoexcitation using light spanning from 280 to 430 nm with an emission Φ_F_ value of 10.2%. Furthermore, tetrahedral **6** was responsive toward I^−^ and Cu^2+^. I^−^ behaved as a fluorescence quencher for **6** through the formation of H-bonding, which locked the cavity of **6** and thus hindered the exchange of the endohedral OTf^−^ counterions. In contrast, owing to the weak cation–π interaction between triazine rings of ligands and Cu^2+^, Cu^2+^ enhanced the emission intensity by disturbing the ILCT process.

### Construction of host–guest systems

The cavity-cored nature of self-assembled metallacages endows them with guest-binding properties. By encapsulating guests with inherent photophysical properties, light-emitting supramolecular systems are obtained, wherein the metallacage provides a confined environment distinct from the bulk solution, leading to the emergence of novel photophysical phenomena. Careful design is often required to avoid undesirable interactions, both spatially and electronically, that quench the emission of light from the system. Fujita and coworkers designed an efficient light-emitting host–guest system (Φ_F _= 17%) comprising the electron-deficient guest fluorophore **8** and metallacage **7** based on Pt(II) and triazine ligands [30]. The electron-deficient nature of **8** avoided the transfer of energy to the metallacage **7**, thus preserving the light-emitting character. Moreover, the host–guest complex was found to be highly soluble in water and to prevent the aggregation of **8**, hence preventing the quenching of its emission by excited-state interactions. This pioneering work revealed the potential of metallacages to serve as hosts for fluorescent dye molecules. The work also paved a way to use metallacage-based host–guest systems for designing smart fluorescent materials.

To prepare supramolecular systems that emit light of various colors and with high efficiency, Yoshizawa and colleagues developed a host–guest system consisting of the Pt(II)-based metallacage **10** and various fluorescent dyes [31]. Their pioneering work demonstrated that the metallacages composed of an anthracene-cored ligand and Ni(II) or Zn(II) exhibited weak to strong emissions of blue light [34]. Based on this result, a range of well-known hydrophobic fluorescence dyes, including a BODIPY derivative (**11**), coumarin 153 (**12**) and Nile red (**13**), were chosen as guest molecules, and the host–guest encapsulation behaviors in water were examined. The resultant aqueous solution of **10**⊃**11** showed strong green fluorescence with a high Φ_F_ value of 48%, while **10**⊃**12** and **10**⊃(**13**)_2_ showed bluish-green (Φ_F_ = 25%) and red fluorescence (Φ_F_ = 32%), respectively. Moreover, the emission behaviors of **10**⊃**11** could be finely tuned upon co-encapsulation of anthracenes, phenanthrene and pyrenes. This study represented a new approach for modulating the emission profile of metallacage-based host–guest systems [46].

## METALLACAGES DISPLAYING AIE PROPERTIES

### Structural chemistry

Tetraphenylethylene (TPE) and its derivatives can be easily synthesized and can also be modified in various ways, and are hence widely used in constructing light-emitting materials with AIE properties [47,48]. In addition, to induce compact aggregation of the TPE molecules, various strategies have been developed to restrict the intramolecular motions of TPE molecules and thus activate their fluorescence emission. Covalent modifications of the TPE structure, such as those accomplished by adding bulky chemical groups and performing cyclization reactions, are extensively used to constrain the intramolecular motions [49]. Alternatively, noncovalent interactions such as hydrogen bonding, host–guest interactions, hydrophobic/hydrophilic interactions and coordination interactions have also been employed [50–52]. Compared to other self-assembly approaches that often generate polymeric materials with limited solvent processability, such as metal–organic frameworks, the self-assembly of metallacages provides structural rigidity that impedes the intramolecular motions of embedded AIEgens, yet yields a retention of good solubility due to their discrete nature. This unique feature allows for solvent-phase characterization and solution-based modularity over photophysical properties.

The structures of representative AIE-active self-assembled metallacages are summarized in Table 2. Initial studies in this area focused on examining their ‘turn-on’ luminescence in both solutions and aggregated states and their levels of responsiveness toward different solvents. Stang and coworkers designed and prepared a poly(ethylene glycol) (PEG)-decorated tetragonal prismatic platinum(II) metallacage **17** driven by the [2 + 4 + 8] self-assembly of tetra-(4-pyridylphenyl)ethylene (TPPE) **14**, PEG-decorated dicarboxylate ligand **15** and *cis*-(PEt_3_)_2_Pt(OTf)_2_**16** [19]. The multi-pyridyl ligand TPPE **14** was observed to not emit light in CH_2_Cl_2_ due to the free intramolecular motions of the TPPE group. However, embedding the nonfluorescent ligand into the rigid metallacages turned on the bright yellow fluorescence with a Φ_F_ value of 10.8% in the CH_2_Cl_2_ solution. This sharp contrast between the emission behaviors of free TPPE ligands and metallacage **17** indicated that immobilizing the group within the metallacages is an efficient way to suppress the intramolecular motions, thus triggering the fluorescence. Moreover, as the relative amount of hexane in the solution was increased, the metallacage **17** gradually aggregated, and the Φ_F_ value continuously increased from 10.8% to 51.3%, accompanied by a change in the emission color from yellow to pale blue. This observation suggested that metallacage **17** remained AIE-active, hence preserving emission behavior at high-concentration regimes through further restrictions on the conformational changes of **14** as a result of the aggregation. In addition, the decorated PEG chains were observed to endow metallacage **17** with high levels of solubility in a wide range of solvents from the relatively apolar CCl_4_ to the highly polar MeOH and water, leading to diverse visible emission colors. Notably, metallacage **17** exhibited an unusual emission of white light upon partially aggregating at room temperature in tetrahydrofuran. The coordinates (0.313, 0.344) for this light in the CIE diagram were found to be close to those of pure white light (0.333, 0.333). The underlying mechanism of this partial AIE of white light from the discrete metallacages differs from that of conventional white-light-emitting materials, which in most cases require a mixture of two or more components. As a result, this scheme complements the current design for white-light-emitting materials and represents a promising approach for developing advanced optoelectronic materials. Overall, TPE-based metallacages with efficient emissions at both low and high concentrations and facile fluorescence tunability were demonstrated, which provides an emerging platform for light-emitting materials with modular photophysical properties.

**Table 2.** Constituent building blocks and cartoon representations of AIE-active light-emitting metallacages.

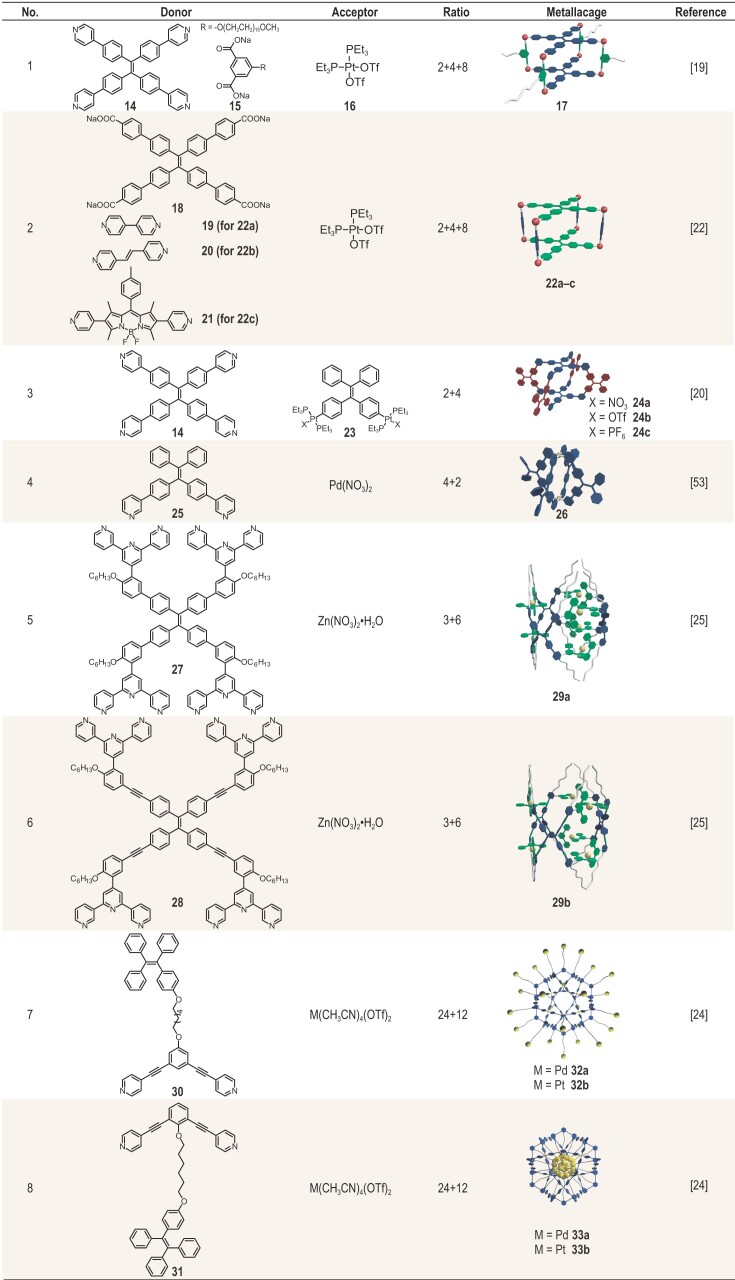

The almost limitless modularity and tunability of the metallacage structures without significant synthetic penalty enable the incorporation of TPE with diverse structures into metallacages. Zhang and coworkers prepared highly emissive tetragonal prismatic metallacages **22a**–**c** by carrying out a coordination-driven self-assembly of benzoate-decorated TPE-core donors **18**, platinum(II) acceptors **16** and three different dipyridyl pillars **19**–**21** [22]. Metallacages **22a** and **22b** showed strong emission bands centered at 493 nm, which originated from the TPE units, while **22c** displayed two such bands, at 472 and 544 nm, which were assigned to TPE and BODIPY groups, respectively. All three of these metallacages strongly emitted light in polar aprotic solvents such as dimethyl sulfoxide (DMSO), with the highest Φ_F_ values of 25%, 20% and 13%, respectively, suggesting that their emission behavior could be precisely tuned by altering the dipyridyl building block.

Instead of integrating dicarboxylate- or dipyridyl-containing ligands as pillars for metallacages, a series of drum-shaped metallacages with different counter anions (NO_3_^−^, OTf^−^ and PF_6_^−^) were achieved by employing both AIE-active donor and acceptor building blocks [20]. Two TPE-core ligands **14** adopted a cofacial arrangement, while the eight exterior freely rotating phenyl rings of four TPE-based organoplatinum donors **23** aligned around the prismatic core. Both donor and acceptor building blocks exhibited weak levels of fluorescence in CH_2_Cl_2_ solution. In comparison, structurally similar but counter anion diverse metallacages **24a**–**c** displayed enhanced levels of emission relative to those of the precursors in dilute CH_2_Cl_2_ solution with the Φ_F_ values of 1.79%, 3.07% and 3.36%, respectively, which could be assigned to the locking of nonemissive TPE ligands within the rigid metallacages. Moreover, upon modular aggregation, the emission intensities increased considerably as the hexane fraction was gradually increased to 90% in the mixed solutions, with the quantum yields reaching 8.87%, 10.6% and 10.9% respectively. Scanning electron microscopy analysis of the aggregates in mixed solutions containing CH_2_Cl_2_ and hexane in a 1 : 9 ratio revealed well-defined nanospheres with average diameters between 150 and 250 nm. Therefore, the compact aggregation of these metallacages restricted the free intramolecular motions of the TPE moiety, explaining the aggregation-induced enhanced emission phenomena.

In addition to the tetragonal prismatic metallacage structures, a lantern-type emissive TPE-based metallacage **26** was synthesized by Sun and colleagues through a [4 + 2] self-assembly of ditopic TPE-based pyridinyl ligand **25** and Pd(NO_3_)_2_ [53,54]. These metallacages showed negligible emission in DMSO, probably resulting from the quenching effect induced by palladium and the nitrate counterion. However, upon addition of toluene, a rather inadequate solution for **26**, the fluorescence of **26** showed an ∼10-fold increase in intensity with a redshift of the maximal emission, indicating its AIE properties and the change of ligand conformation during aggregation. Moreover, the fluorescence of **26** can be turned on by encapsulating HCO_3_^−^ anions in its cavity, indicating an encapsulation-induced emission behavior. The formation of strong hydrogen bonding between the encapsulated anions and the inner surface of **26** was responsible for restricting the motions of the otherwise freely rotating TPE units, thus inducing their emission behavior.

Multiple luminophores can be introduced into a single metallacage system to induce sophisticated photophysical behaviors that originate from their interactions. Wang and coworkers described another elaborate design of light-emitting metallacages that integrated TPE and terpyridine (tpy) as ligands [25]. The final two types of metallacages, **29a** and **29b**, formed via the coordination-driven self-assembly of three TPE-based tpy ligands (**27** or **28**) and six Zn(II) ions. Compared to **29a**, the constituent ligands of **29b** contained an additional alkyne unit between the TPE and tpy units, increasing the ligand rigidity and extending the conjugation of the structure. The fluorescence spectrum of ligand **27** showed a single emission band centered at ∼425 nm, which stemmed from the tpy units. In contrast to ligand **27**, ligand **28** displayed two such bands, peaking at 430 and 550 nm, ascribed to the tpy and TPE units, respectively, suggesting that the ligand with enhanced rigidity efficiently suppressed the rotation of the TPE units and thus triggered their AIE behavior. After coordinating with Zn(II), cages **29a** and **29b** each showed only a single emission band centered at 510 nm, attributed to TPE units. The emission of light from the tpy units was quenched because of the nonradiative decays through an MLCT process wherein the excited-state energy was transferred to the Zn(II) centers. The quantum yield of metallacage **29b** in dilute acetonitrile solution (1.00 μM) reached 20.79%, 4.5-fold higher than that of **29a** (4.67%) under the same conditions. This highly light-emitting nature of metallacage **29b** originated from the structural rigidity that limited the rotation of TPE units in discrete modules rather than having originated from partial aggregation, hence representing a new strategy to exploit metallacages with strong emissions in dilute solutions.

The above examples describe the light-emitting metallacages prepared by locking the AIEgens within their rigid skeletons to constrain their intramolecular rotation, turning on their fluorescence. Besides this approach, another strategy inspired by the green fluorescent protein was designed to realize light-emitting metallacages by anchoring AIEgens within the confined cavities of metallacages to turn on their fluorescence [24]. Through coordination-driven self-assembly, 24 TPE units were precisely distributed either on the outer surface of each of metallacages **32a** and **32b** by *exo*-functionalizing the ligands or within the inner confined space of each of **33a** and **33b** by performing *endo*-functionalization. The absorption profiles showed lower absorption bands for the Pt_12_L_24_ self-assemblies than for their corresponding ligands, but similar intensities of the bands for the Pd_12_L_24_ self-assemblies and their corresponding ligands. This distinction may stem from the enrichment of the π-system by the comparatively strong π backbonding from the Pt center to the nitrogen π^*^, which lowers the excitation energy of donors in the Pt_12_L_24_ self-assemblies. Regarding their emission properties, both *endo*- and *exo*-functionalized metallacages showed fluorescence enhancements compared to their constituent ligands, attributed to the locally increased concentration of the TPE units. However, suffering from strong MLCT, the Pd_12_L_24_ metallacage displayed a lower fluorescence enhancement than its Pt analogs. Notably, in dilute solution, the emission of the endohedral functionalized metallacage **33b** was found to be much stronger than that of the exohedral metallacage **32b**. The endohedral functionalization induced a much higher local concentration of the 24 TPE units in the confined cavities than did the exohedral functionalization, and resulted in more restrictions on the intramolecular rotations of the TPE units and thus strengthened the emission.

### Factors that influence the emission properties of AIE-active metallacages

The structural diversity and facile tunability of metallacage structures enable the systematic investigation of the factors that affect their photophysical profiles, including emission wavelength and intensity. These factors include external ones, such as solvents, temperature and pressure, as well as internal ones stemming from the design of the metallacages, such as structural rigidity, counterions and interactions with other components in the metallacage.

#### Solvent

**Figure 1.**
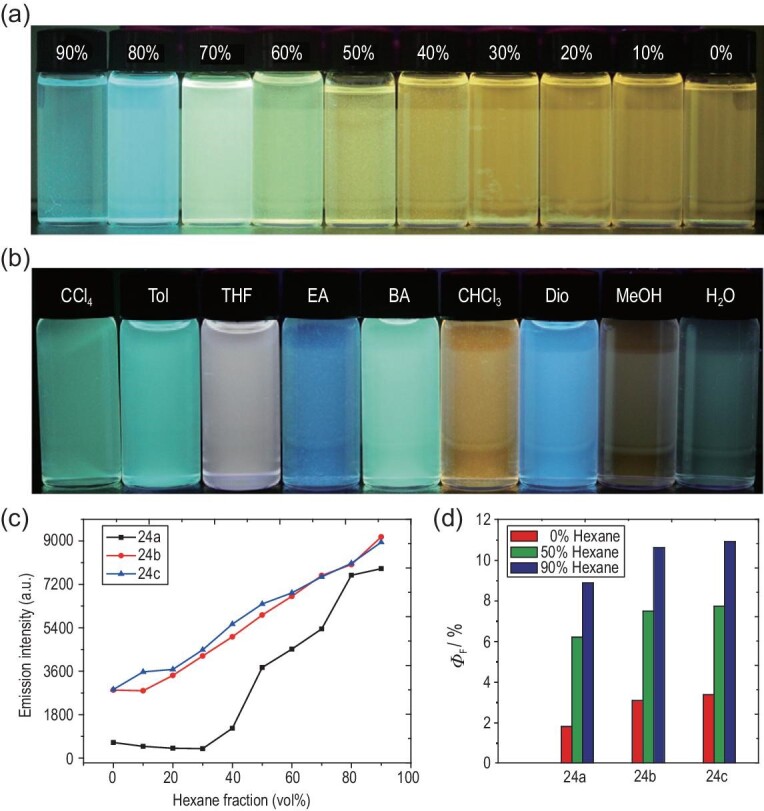
(a) Photographs of metallacage **17** in CH_2_Cl_2_/hexane mixtures with different fractions of hexane on excitation at 365 nm and 298 K (*c* = 10.0 μM). (b) Photographs of metallacage **17** in different solvents: Tol, toluene; EA, ethyl acetate; BA, butyl acetate; Dio, dioxane. Adapted with permission from [19]. (c) Plots of maximum emission intensity of **24a–c** versus hexane fraction in CH_2_Cl_2_/hexane mixtures on excitation at 365 nm and 298 K (*c* = 10.0 μM). (d) Quantum yields of **24a–c** versus hexane fraction in CH_2_Cl_2_/hexane mixtures. Adapted with permission from [20].

The solubility of an AIE metallacage in a particular solvent determines the physical states of the metallacage, and thus significantly influences its light-emitting properties. In general, addition of poor solvents leads to aggregation of metallacages, which can be visualized using microscopic techniques [19,20]. For instance, the average *D*_h_ of metallacage **17** increased from 12.54 to 255 nm upon increasing the hexane content to 40%. Further elevating the hexane content to <60% caused the aggregation of **17** into nanoparticles, which then evolved into regular nanospheres for hexane contents >60% [19]. These morphology changes represented a tighter modular packing, imposing further restrictions on the conformational changes that can be made by the TPE moieties compared to the discrete rigid metallacages. As a result, the emission increased in intensity and was blueshifted (Fig. 1a). At the same time, the Φ_F_ value at a hexane content of 90% was nearly 4.8-fold higher than that of the discrete entity in pure CH_2_Cl_2_, suggestive of their inherent AIE properties. An enhancement was also observed for **33b** [24]. Increasing the hexane content in the CH_2_Cl_2_ solutions of these metallacages gradually enhanced the fluorescence, which was caused by the further suppression of the intramolecular motions of these TPE units upon their having undergone solvent-induced aggregation.

In addition to the molecular aggregation caused by poor solvents, the different conformations adopted in different solvents could vary the degree of conjugation of the TPE motifs, thereby changing the emission wavelength and intensity. Liu and coworkers observed that when gradually adding water to the acetone solution of metallacage **17** (but replacing eight OTf^−^ groups with eight NO_3_^−^ groups to increase the solubility of the metallacage in water and acetone), a stronger emission and a blueshift in the emission maximum occurred [55]. These changes are attributed to the hydrophobicity of TPPE ligands, which become twisted in water, reducing their degree of conjugation.

The solvent polarity is another key factor affecting the photophysical properties of AIE-active metallacages because of its influence on the many energy transfer processes between the components of the metallacages, especially those involving the transfer of charge between AIEgens and the metal centers. For example, metallacage **17** showed irregular differences in emission intensities and maximal emission wavelengths between solvents ranging from the relatively apolar CCl_4_ to the highly polar MeOH and water (Fig. 1b) [19]. A comprehensive investigation revealed the mechanism behind these changes: the solubility of metallacage **17** in a specific solvent controls the photophysical properties. When the different solvents equally well dissolve **17**, the solvent polarity is the dominating factor for the light-emitting behavior, due to its influence on the MLCT processes within the metallacages. In this way, the higher polarity leads to a stronger MLCT process, thereby providing poorer fluorescence efficiency.

However, altering the building blocks of metallacages alters the fluorescence response to solvents of different polarities. Regarding metallacages **22a**–**c**, they all were observed to exhibit strong emissions in polar aprotic solvents (DMF, DMSO and acetonitrile), but moderate ones in less polar aprotic solvents (acetone, CH_2_Cl_2_ and CHCl_3_) as well as a polar protic solvent (MeOH) [22]. Although the two cases above were concluded to afford different fluorescent changes and mechanisms with respect to solvents of different polarities, it was confirmed that the emission properties of TPE-based metallacages can be efficiently tuned by changing the solvent.

#### Temperature

Temperature affects the intramolecular motions of TPE units and changes the fluorescence intensity stemming from their AIE behavior. In general, lowering the temperature of a TPE-based metallacage solution significantly limits the rotation of the TPE motifs, reducing the nonradiative decay and enhancing the emission of light. For example, when freezing acetonitrile solutions of **29a** and **29b** with liquid nitrogen to 77 K, their fluorescence intensities both increased [25]. However, a gradual increase in temperature was observed to lead to a decrease in the emission intensity of **17**, but to no change in the emission wavelength. The partial dissociation of the metal-coordination bonds at elevated temperatures might be responsible for the weaker fluorescence [19,26].

#### External hydrostatic pressure

One prominent feature of TPE-based metallacages is their strong emission in the solid state, with this feature attributed to the full inhibition of intramolecular motions. However, upon imposing an external hydrostatic pressure, the molecular packing changes and alters the fluorescence behaviors [26]. For metallacage **29b**, its emission intensity increased as the pressure was increased from 0 to 1.10 GPa, but became quenched as the pressure was gradually increased further to 6.32 GPa [25]. Hence, increasing the pressure at low-pressure ranges may further suppress the intramolecular motion of the TPE units, but excessive pressures eventually lead to quenching because of the enhanced dipole–dipole interactions. This mechanical response of emission properties opens a promising route to creating highly emissive materials for applications as mechanochromic sensors.

#### Structural rigidity

The structural rigidity of an AIE-active metallacage is a major internal factor that influences its photophysical behavior [56]. For example, benefiting from the enhanced ligand rigidity resulting from introducing an additional alkyne group into **28**, metallacage **29b** showed a stronger fluorescence in dilute solutions than **29a** [25]. Moreover, by mixing ligands **27** and **28** to fabricate hybrid metallacages, a series of assemblies with different ratios of **27** to **28** were furnished and followed a statistical distribution. The fluorescence profiles suggested the stronger emissions from the metallacages predominantly made up of **28**, verifying the greater favorability of a higher degree of structural rigidity for the fluorescence of TPE motifs.

Another strategy for imposing a higher degree of structural rigidity is to shorten the distance between discrete metallacages in the aggregated state, where the TPEE units are fixed in a twisted, less conjugated state and exhibit higher energy emissions [57].

#### Anion effects

In most cases, self-assembled metallacages are cationic in nature due to the use of metal ions or organometallic complexes as structural components. As a result, anions are necessary to balance the overall charge of the metallacage. Anions have been observed to significantly affect the solubility levels of SCCs, and in some cases even determine the structural outcome of the self-assembly. In the aggregated states, anions also influence the packing of supramolecular ensembles. For light-emitting metallacages, the effect of anions on the photophysical properties was examined. The fluorescence profiles for metallacages **24a**–**c** (Fig. 1c), made of the same cage structure but different anions (NO_3_^−^, OTf^−^ and PF_6_^−^), were investigated [20]. Although all of them displayed typical AIE behavior in a CH_2_Cl_2_/hexane mixture, the molar absorption coefficients, fluorescence emission intensities and quantum yields of these metallacages followed the order of PF_6_^−^ > OTf^−^ > NO_3_^−^ (Fig. 1d). This phenomenon is attributed to the difference between the solubilities of the cages as a result of different counterions they have in their structures.

### Applications of AIE-active metallacages

The versatile chemical designs of ligands have enabled researchers to produce structurally complex and functionally diverse metallacages. Regarding AIE-active fluorescent metallacages, extensive efforts have been devoted to developing such metallacages with novel photophysical properties and exploiting their potential applications as chemical sensors [19,22,25], functional emissive materials [22,58–60], light-harvesting systems [61,62] and theranostic agents [21,63,64]. In this section, the development and advances in the applications of AIE-active metallacages in diverse states ranging from discrete entities to multidimensional supramolecular materials are highlighted.

#### Sensors

Luminescence is a readily available spectroscopic approach for sensing applications. The fluorescence ‘turn-on’ behavior of many AIEgens provides the basis for their sensing applications, and incorporating AIEgens into metallacages not only preserves their desirable photophysical features but may also induce novel responsiveness due to the interplay between AIEgens and constituent building blocks [19,26,54]. For example, **17** exhibited a distinct emission color in the visible region when dissolved in structurally similar esters [19], and this emission was attributed to the inherent aggregation behaviors and charge transfer properties of **17** in the different solvents.

**Figure 2.**
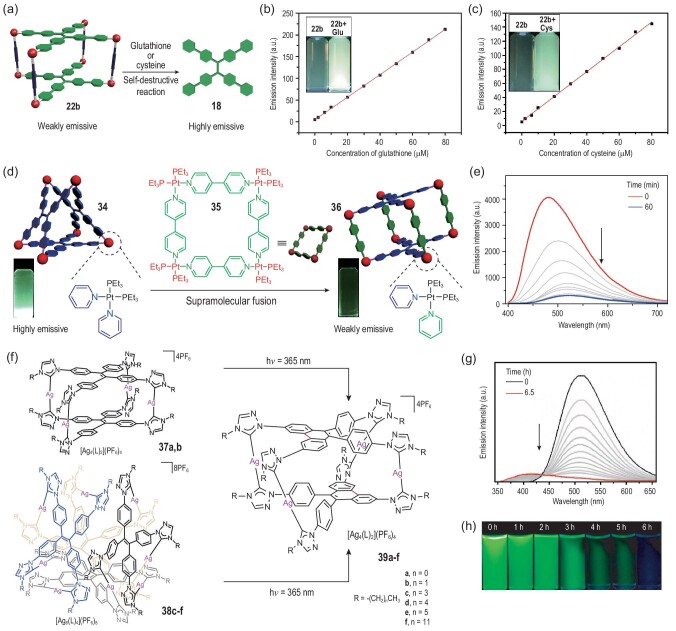
(a) Cartoon representation of the self-destructive reaction for amino acid sensing. The emission intensities at 500 nm of **22b** after 5 min upon the addition of increasing concentrations of (b) glutathione (Glu) and (c) cysteine (Cys) as a function of glutathione and cysteine concentrations. Adapted with permission from [22]. (d) Cartoon representation of the supramolecular fusion process and (e) time-dependent changes in the emission profiles of the mixture of **34** and **35** (2 : 3 molar ratio) in DMSO. Adapted with permission from [65]. (f) The metal-coordination self-assembly processes and the photoinduced supramolecule-to-supramolecule transformations. (g) Fluorescence emission spectra and (h) photographs of **38c** after UV irradiation for different periods of time. Adapted with permission from [66].

Apart from mechanisms involving the sensitivity of metallacages to solvent solubility and polarity, a mechanism involving a self-destruction of a metallacage was reported to operate for its sensing of amino acids (Fig. 2a). Since metallacage **22b** is nearly nonemissive in MeOH/water (1/1, *v/v*), it can be used as a turn-on fluorescence sensor for thiol-containing amino acids through a self-destructive reaction [22]. The Pt–N coordination bonds of the metallacage can be disrupted by adding thiol-containing amino acids to the metallacage due to the Pt–S bonds they would form in this case being stronger than the relatively dynamic Pt–N bonds. Such a reaction was shown to release a TPE-based benzoate acid building block, which is highly emissive in MeOH/water (1/1, *v/v*) because of its poor solubility in this mixture. Specifically, upon gradual addition of glutathione or cysteine, the emission intensities linearly increased with the amino acid concentration in the range 0–80 μM (Fig. 2b and c), with detection limits of 1.87 × 10^−7^ and 2.78 × 10^−7^ M (S/N = 3) for glutathione and cysteine, respectively. Furthermore, the metallacages can be regenerated by further adding Pt(II) acceptors. Therefore, metallacage **22b** can be used as a ‘turn-on’ sensor for amino acids. Such an interesting case provides a stimuli-responsive destruction mechanism and endows the cages with great potential for applications in controlled drug release and other bio-related systems.

#### Monitoring the transformations of supramolecular structures

Inspired by natural processes such as molecular recognition, signal transduction and allosteric regulation, scientists are constructing synthetic supramolecular systems to mimic the biomolecular transformations with the aim of elucidating the mechanisms of these processes as well as fabricating materials with functions similar to those of biomolecules. However, the complexity of supramolecular systems presents a significant challenge for efficiently tracking their transformation processes in real time. Therefore, it has been considered highly desirable to develop a method

to address the issue. For this purpose, Yan and coworkers proposed an efficient supramolecular transformation system: a TPE-containing trigonal prism **34** and its supramolecular fusion with a two-component molecular square **35** into a three-component molecular metallacage **36** (Fig. 2d) [65]. The entire process can be readily monitored by measuring the changes in the fluorescence signals of the supramolecular systems, due to the trigonal cage **34** exhibiting strong fluorescence via the restriction of TPE intramolecular motions upon coordination, but the transformation to the three-component cage as a result of heteroligation of the platinum center partially quenching the fluorescence as well as the redshifts in the emission maximum (Fig. 2e). According to the theoretical computations, the observed fluorescence quenching was attributed to the photoinduced electron transfer between the TPE and 4,4^′^-bipyridine motifs as the lowest unoccupied molecular orbitals of the cage **36** are located on the bipyridine rings. Therefore, the results demonstrated the feasibility of real-time tracking of the supramolecular cage-to-cage transformation via the AIE phenomenon. This work showed an elegant synergy between AIE and supramolecular chemistry and can be used to provide unique avenues and perspectives for the efficient design, tracking and characterization of complex, dynamic supramolecular systems with favorable photophysical properties for broad applications.

Recently, Han and coworkers explored the coordination chemistry of TPE-bridged tetrakistriazolylidene ligands and Ag metal centers as building blocks for the construction of supramolecular tetranuclear ([Ag_4_(L)_2_](PF_6_)_4_) **37a**–**b** and octanuclear ([Ag_8_(L)_4_](PF_6_)_8_) metallacages **38c**–**f** (Fig. 2f) [66]. The metallacages showed enhanced fluorescence due to the restriction of the TPE motifs upon coordination bond formation. Upon being exposed to UV irradiation in air, **37a**–**b** and **38c**–**f** underwent oxidative photocyclizations at the tetrakisarylethylene unit to yield complexes **39a**–**f**, respectively. Note that all six of the products, i.e. **39a**–**f**, shared the same tetranuclear metallacage structural plan despite the overall structures of the reactants **37a**–**b** being quite different from the reactants **38c**–**f**. These products **39a**–**f** all included the 9,10-phenyl-substituted phenanthrene bridge, a feature responsible for immobilizing the tetrakistriazolylidene ligands and thereby quenching the fluorescence. Hence, due to the nonemissivities of the resulting metallacages, this *in situ* conformational transformation was monitored by having the fluorescence tracked, where the fluorescence continually weakened with prolonged irradiation time until it became fully quenched within 6 h (Fig. 2g and h).

#### Multidimensional light-emitting materials

The above examples presented the promising fluorescence properties of the AIE-active metallacages in the discrete state. Given their unique photophysical properties, TPE-based metallacages have been regarded as building blocks for the fabrication of multidimensional superstructures or supramolecular soft materials with fluorescence tunability. These features have paved the way to exploiting highly emissive light-emitting materials. Sun, Stang and colleagues reported such complex architectures with multicolor emissions based on the TPPE-core metallacages [59]. The metallacages were furnished by combining dicarboxylate ligand derivatives with various substituents (sodium sulfonate, nitro, methoxyl and amine), TPPE and *cis*-(PEt_3_)_2_Pt(OTf)_2_. Finally, the obtained metallacages were used as building blocks and further assembled to form 1D, 2D and 3D superstructures with broad emission wavelengths (*λ*_max_ ranging from 451 to 519 nm) and tunable fluorescence features [60]. By using the ‘phase-transfer’ co-assembly process, lysine-modified perylene was incorporated into metallacage-based microflowers, broadening the library of light-emitting superstructures. Similarly, chlorophyll a and vitamin B_12_ were introduced into the microflowers during the assembly process, and such procedures might be exploited in studies of energy capture and nerve repair in the future.

**Figure 3.**
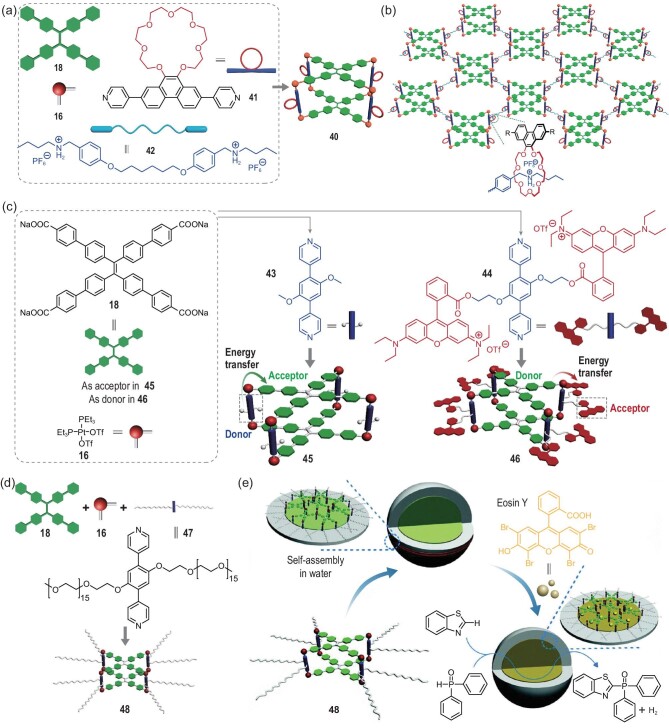
(a) Self-assembly of 21C7-decorated metallacage **40** and (b) the formation of cross-linked supramolecular polymer network from metallacage **40** and bisammonium salt **42**. Adapted with permission from [23]. (c) Self-assembly of metallacages **45** and **46** with reverse FRET behavior. Adapted with permission from [26]. (d) Self-assembly of the metallacages **48** and (e) the formation of the artificial light-harvesting system with **48** and its application in a photocatalytic reaction. Adapted with permission from [61].

At the same time, the integration of fluorescent metallacages into supramolecular gels has attracted significant attention due to the well-defined hierarchical order and tunable emission properties of the resultant materials [58]. Yin and coworkers designed a fluorescent metallacage-cored supramolecular gel by achieving orthogonal metal–ligand coordination and host–guest interactions between the 21-crown-7 (21C7) and dialkylammonium salts [23]. The crown-ether-decorated tetragonal prismatic metallacage **40** was prepared by carrying out a metal-coordination-driven self-assembly of **18**, **16** and linear dipyridyl ligand **41** (Fig. 3a). Subsequent addition of a bisammonium linker to **42** delivered a supramolecular polymer network (Fig. 3b). As a result, and specifically due to the incorporation of metallacages with AIE properties, the gel was highly emissive. Responsiveness to multiple stimuli, involving heat and addition of KPF_6_, and good self-healing properties were also observed here because of the dynamic nature of the metal coordination and host–guest interactions. Furthermore, the gel with metallacage cores displayed 10-fold higher storage and loss moduli than that without cores, indicating that the rigid metallacage enhanced the stiffness of the gel. This work not only enriched the functionalization approaches of fluorescent metallacages, but also paved the way for preparing dynamic yet robust supramolecular light-emitting materials.

#### Light-harvesting system

Photosynthesis is widespread in living systems for the conversion of light energy to chemical energy. Fluorescence resonance energy transfer (FRET) represents the energy transfer behavior between different chromophores through a nonradiative dipole–dipole coupling pathway. An efficient FRET system requires a favorable distance between energy donors and acceptors, and a good overlap in

the fluorescence spectra between the emission of energy donors and the absorption of acceptors. In this regard, metallacages are good candidates for investigating FRET to mimic the natural energy harvesting process, due to their well-defined structures that precisely organize the constituent luminophores. Zhang *et al.* prepared two metallacages comprised of TPE-core benzoate **18** and two different dipyridyl pillars **43** and **44** by carrying out a coordination-driven self-assembly (Fig. 3c) [26]. Compared to pillar **43**, **44** was functionalized with rhodamine B. The photophysical characterizations revealed a good overlap between the absorption band of **18** and the emission band of ligand **43**. As a result, metallacage **45** exhibited a FRET from the **43**-containing pillar building blocks to **18**. In contrast, cage **46** showed FRET from **18** to the pillar **44**, due to the adequate spectral overlap between the emissions of **18** and **44**. Due to **45** and **46** being inherently emissive in dilute solution as a result of the restriction on the motions of the TPE-based ligands within the rigid skeletons, the reverse FRET process within the two cages led to distinct photophysical properties, which derived from the different solubilities as well as the distinct FRET efficiencies of the solvents. As such, these investigators demonstrated an efficient FRET system for metallacages wherein the fluorescence was finely tuned by altering the FRET behavior, providing a promising platform for developing artificial light-harvesting systems via mimicking the natural photosynthesis process. Therefore, they further constructed an artificial light-harvesting system in water based on FRET by making use of the light-emitting PEG-decorated tetragonal prismatic Pt(II) metallacage **48** as the energy donor molecule and eosin Y as the energy acceptor [61] (Fig. 3d). Note that eosin Y is a well-known photosensitizer used to catalyze C–H arylation of heteroarenes and cross-coupling. Metallacage **48** could self-assemble into micelles because of the hydrophilicity of the lateral PEG chains and hydrophobicity of the internal cavities of the metallacage (Fig. 3e). Because of the rigidness of the TPE units, metallacage **48** displayed a strong emission at 511 nm with a 23.8% quantum yield; this emission showed good spectral overlap with the absorption of eosin Y. As a result, an efficient light-harvesting system was achieved. This light-harvesting system exhibited a higher photocatalytic activity for the cross-coupling hydrogen evolution reaction than did eosin Y alone because of the additional utilization of light energy in the UV regions by metallacage **48** and the subsequent activation of eosin Y through the FRET process. This work represented an elaborate design of metallacages with complex structures and functions, and opened the avenue to constructing artificial photosynthesis systems with highly emissive TPE-cored metallacages.

#### Targeted theranostic agents

So far, platinum-based compounds such as cisplatin, oxaliplatin and carboplatin have constituted a mainstay of clinical chemotherapeutics used to treat many solid tumors. However, severe side effects, poor specificity and an inability to track the process of translocation have called for new agents that combine a targeting ability, diagnostic capabilities and therapeutic functions. AIE-active metallacages show excellent potential as theranostic agents because metal ions, which are necessary elements in their structure, provide anticancer effects. Moreover, the high fluorescence efficiency levels of AIE-active metallacages in various environments allow for facile tracking and imaging [64] of their activities in biological systems.

**Figure 4.**
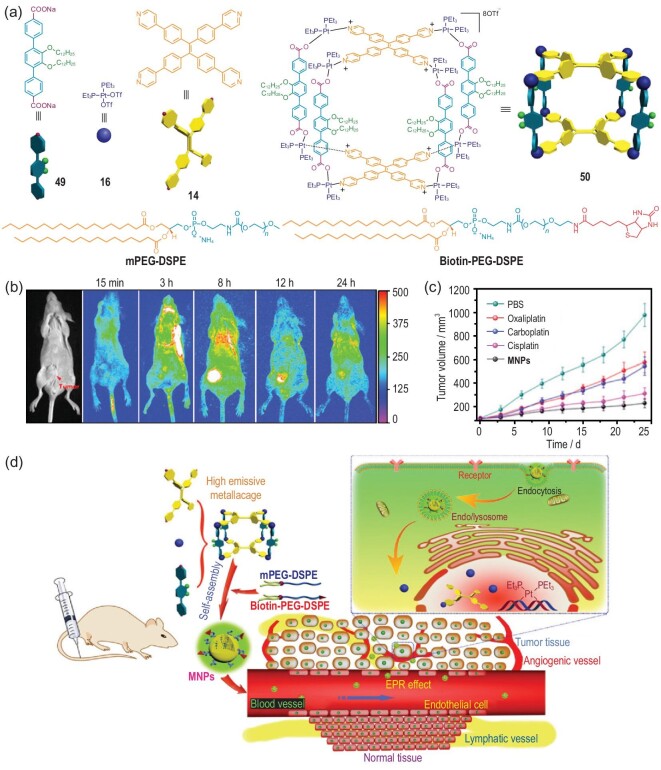
(a) The self-assembly of metallacage **50** and the mPEG–DSPE and biotin–PEG–DSPE agents used for MNPs’ formation. (b) *In vivo* fluorescence imaging of nude mice bearing HeLa cancer xenograft following *in vivo* injection of MNPs. (c) *In vivo* tumor growth inhibition curves for phosphate-buffered saline, oxaliplatin, carboplatin, cisplatin or MNPs on the HeLa tumor model. (d) Scheme of MNPs’ transportation within blood vessels and accumulation in tumor tissue, followed by receptor-mediated endocytosis. Adapted with permission from [21].

By carrying out multicomponent coordination-driven self-assembly, Huang, Stang and coworkers prepared the highly emissive TPE-based metallacage **50** and utilized it as a theranostic platform for cancer therapy (Fig. 4a) [21]. Two variants of the 1,2-distearoylphosphatidylethanolamine/PEG conjugate, mPEG–DSPE and biotin–PEG–DSPE, were used to form nanoparticles embedded in the metallacage to improve its circulation time and accumulation in tumors by taking advantage of the enhanced permeability and retention (EPR) effect. *In vitro* studies have shown the metallacage-loaded nanoparticles (MNPs) exhibiting excellent targeting capability and selectively delivering MNPs to biotin-receptor-overexpressing cancer cells via receptor-mediated endocytosis. *In vivo* fluorescence imaging (Fig. 4b) identified strong fluorescence signals that originated from the highly emissive MNPs in tumor tissues, confirming their diagnostic capability. Moreover, as shown in Fig. 4c, *in vivo* experiments demonstrated that the MNPs exhibited higher antitumor efficacy with lower toxicity than did free platinum anticancer drugs (oxaliplatin, carboplatin and cisplatin), attributed to their EPR effect and active targeting ability (Fig. 4d).

Apart from physical encapsulation into polymeric nanocarriers, modification of ligands for achieving specific recognition is another effective way to endow metallacages with targeting capacities. By introducing extra biotin moieties into the pillars of TPE-containing metallacages, Zhang and coworkers developed a light-emitting metallacage as a cancer-targeting theranostic that was specifically processed to selectively target cancer cells that overexpress biotin receptors [63]. Owing to the presence of biotin receptors on the HepG2 cells, metallacages with eight biotin groups easily attached to HepG2 cells, which further enhanced their endocytosis, as shown by the bright green fluorescence inside the cells. An MTT assay revealed that although their levels of cytotoxicity toward cancer cells were lower than those of the commercial anticancer drug cisplatin, they displayed a higher selectivity toward cancer cells that overexpress biotin receptors.

## SUMMARY AND OUTLOOK

In this review, the recent achievements in the field of light-emitting metallacages have been summarized, including the principles for their rational design and efforts to explore their applications. The well-defined, tunable structures of metallacages have provided a versatile platform for taking advantage of their photophysical properties. Notably, combining metallacage chemistry with AIE has led to the development of AIE-active metallacages displaying favorable photophysical properties such as high luminescence efficiency and good modularity and having impressive relevance to a wide variety of areas such as sensing, energy conversion and the development of theranostic agents [15,67–69].

Nonetheless, many challenges remain, while several aspects are crucial for the future development of light-emitting metallacages. Current examples of light-emitting metallacages mainly involve noble metals and lanthanides. While the use of these metals is beneficial for establishing the scope and limitations of light-emitting metallacages, developing strategies for light-emitting metallacages constructed from abundant metals would be highly desirable for their scale-up and practical applications. Computational methods are expected to play an increasingly prominent role not only for predicting the structural outcomes of the self-assembly reactions but also for investigating the spatial and electronic interactions between the components within the metallacage structures. Systematic investigations of these interactions provide the basis for rationally designing metallacages with sophisticated photophysical behaviors that require a precise and synergistic combination of multiple photophysically active building blocks. Specifically with respect to the AIE-active metallacages, the rapid development of AIE offers continuous inspirations for the use of advanced AIE-active metallacage materials. The use of AIEgens with properties such as multiphoton absorption, red/near-infrared emission, enhanced solubility and biocompatibility—that is, properties more desirable than those of the extensively studied TPE—is expected to result in the development of supramolecular luminophores with broader potentials. Combining AIEgens with metallacage-cored supramolecular polymers might lead to dynamic polymer materials that respond to diverse chemical and physical stimuli by emitting light. The possibilities of using AIEgens in photodynamic, photoacoustic and photothermal therapies would permit an expansion of the range of biomedical applications of AIE-active metallacages. Overall, with the rapid advances of both coordination-driven self-assembly and luminophores with favorable photophysical properties such as AIE, it is expected that research on light-emitting self-assembled metallacages will continue to flourish.
